# Themes, Policies, and Attention Shifts Regarding COVID-19 Vaccinations in German-Speaking Regions: Infoveillance Study Using Tweets

**DOI:** 10.2196/63909

**Published:** 2025-10-31

**Authors:** Katarina Boland, Christopher Starke, Felix Bensmann, Frank Marcinkowski, Stefan Dietze

**Affiliations:** 1 Computer Science Heinrich-Heine-University Düsseldorf Düsseldorf Germany; 2 Knowledge Technologies for the Social Sciences GESIS - Leibniz Institute for the Social Sciences Cologne Germany; 3 Amsterdam School of Communications Research University of Amsterdam Amsterdam The Netherlands; 4 Communication and Media Studies Heinrich-Heine-University Düsseldorf Düsseldorf Germany

**Keywords:** COVID-19, web discourse data, Twitter, vaccination hesitancy, data-driven policy making

## Abstract

**Background:**

Societies worldwide have witnessed growing rifts separating advocates and opponents of vaccinations and other COVID-19 countermeasures. With the rollout of vaccination campaigns, the European German-speaking region (Germany, Austria, and Switzerland) initially exhibited a noticeably low vaccination uptake compared to other European regions. Later, uptake increased. It remains unclear which factors contributed to these changes.

**Objective:**

This study aimed to shed light on the intricacies of vaccine hesitancy among the German-speaking population and the possible dynamics between policy changes and public concerns using web discourse data. These insights are valuable for policymakers tasked with making far-reaching decisions—policies need to effectively curb the spread of the virus and at the same time respect fundamental civil liberties and minimize undesired consequences.

**Methods:**

This study drew on data from Twitter (subsequently rebranded X). We used a hybrid pipeline to detect and analyze 191,750 German-language vaccination-related tweets using a semiautomatic seed list generation approach, topic modeling, sentiment analysis, and a minimum of social scientific domain knowledge to evaluate the discourse about vaccinations in light of the COVID-19 pandemic. We further analyzed the evolution of public attention during different phases of the pandemic and in relation to policy changes to identify potential drivers of shifts in public attention.

**Results:**

The discourse concerning vaccinations was associated with more negative sentiments than the general discourse on German-speaking Twitter (47,159/191,750, 24.59% vs 1,758,776/12,297,163, 14.3% predominantly negative tweets, respectively). The relative frequencies of the discussed themes fluctuated heavily (eg, *safety and side effects* was the most dominant theme in wave 3 [1,611/9,179, 17.55%] but ranked 6th in wave 5 [428/4,865, 8.8%], and *effectiveness of vaccinations* ranked 7th in wave 3 [711/9,179, 7.75%] and 2nd in wave 5 [831/4,865, 17.08%]). In wave 3, vaccines were authorized, and vaccinations were suspended and resumed due to safety concerns. Later, policies were implemented that restricted the freedom of unvaccinated citizens. Change points in attention aligned better with policy actions than with pandemic phases. During the later phases, vaccination uptake increased (wave 2: 5.6%, wave 3: 47%, and wave 5: 74% compared to 30%, 62%, and 78%, respectively, in the United Kingdom), and so did the attention to *freedom and civil liberties* (wave 2: 1,139/6,595, 17.27%; wave 5: 1,403/4,865, 28.84%). Substantially increasing negative and stronger sentiments were expressed.

**Conclusions:**

Our analyses suggest potential interactions among policies, public attention to different topics, and associated sentiments. While vaccination uptake increased, our findings indicate that citizens’ doubts and concerns did not decrease and that, rather than being fully persuaded, they remained skeptical. This study showcases that monitoring web discourse can provide valuable insights for data-driven policymaking in highly dynamic contexts such as the COVID-19 pandemic.

## Introduction

### Background

The outbreak of the COVID-19 pandemic fundamentally disrupted societies worldwide. To protect the public and particularly groups considered vulnerable, governments introduced policies to limit the spread of this infectious disease. These policies included mask mandates, closing schools and the retail sector, curfews, strict lockdowns, and contact restrictions. While many of these measures are assumed to have successfully slowed the spread of the pandemic and contributed to saving lives, they also had detrimental side effects. For instance, a stalled economy led to widespread unemployment [1], and lockdown policies strained people’s mental health and were accompanied by increased domestic violence [2]. This balancing act forced governments to make difficult trade-off decisions in designing policies with maximum effectiveness and minimal invasiveness. COVID-19 vaccination strategies are a prime example of this tightrope walk. On the one hand, there is empirical evidence suggesting that widespread vaccination uptake ranked among the most effective means to protect the population from being infected or hospitalized [3]. Thus, encouraging vaccination uptake was primarily seen as a promising strategy to speed up the reopening of society. However, on the other hand, invasive policies to promote or even coerce vaccine uptake, such as vaccine mandates or limiting rights for unvaccinated citizens, marked a severe encroachment of civil liberties and spurred a significant backlash among the citizenry [4]. For instance, invasive policies increased polarization among citizens, undermining social peace and democracy [5]. Therefore, considering citizens’ concerns is crucial when designing adequate vaccination policies and minimizing negative side effects on society.

In particular, the German-speaking region (Deutschland [Germany], Austria, and CH [Confoederatio Helvetica, Latin for Switzerland]; DACH) in Europe exhibited higher vaccine hesitancy than other European countries at the start of the pandemic [6]. While polling is currently the dominant strategy for governments to retrieve citizens’ attitudes, opinions, and behaviors [7], web-based public discourse is gaining ground as an additional source of information [8-10]. Appropriate data are often retrieved from social networking sites such as Twitter (subsequently rebranded X), Facebook, or YouTube and then analyzed using natural language processing methods. While this type of data has been criticized for its lack of representativeness and oversampling of younger, privileged, and technology-savvy people [11], it also offers several distinct characteristics that distinguish these data from traditional survey data. First, discourse data are highly dynamic and cheap and contain information about billions of users [12]. Second, they can be analyzed ex-post, thereby shedding light on longitudinal fluctuations in public opinion about real-life events [13]. Third, they allow for a more nuanced understanding of the ambivalence in public opinion [14] by providing insights into the discursive construction of contentious issues [15]. Hence, many scholars have argued that survey and discourse data can complement each other [16-19], particularly in highly dynamic policy contexts such as the COVID-19 pandemic. Indeed, attitudes drawn from surveys and web discourse show similar long-term trends, with discourse data sentiments being more prone to short-term fluctuations [20] and polarized opinions about COVID-19 measures being more pronounced in survey data [21]. Especially in times of crisis, continuously monitoring how public opinion changes is crucial for policymakers to make informed decisions. For instance, discourse data can help policymakers assess salient topics and concerns that may lead to vaccine hesitancy, evaluate the strength of sentiments associated with the discourse, or retrieve information about side effects in real time. This may help select appropriate policies. For instance, information campaigns can address citizens’ concerns about potential side effects of the vaccine, whereas tangible incentives can be a suitable means to boost vaccine uptake for free riders.

### Objectives

Our study investigated the potential of web discourse data to inform policymaking about the vaccination strategy in the DACH region. We combined automated methods with manual analyses to harvest and extract suitable data from Twitter. Specifically, we proposed a semiautomatic analysis pipeline consisting of tweet filtering, sentiment analysis, and topic modeling to trace the rapid change in topics and public sentiment in the vaccination discourse. We further shed light on how the evolution of topics related to important policy events outlined in the national vaccination strategies of the 3 countries under investigation. Thus, we formulated the following research questions (RQs):

1. How much attention did the topic of vaccination receive on German-speaking Twitter during the pandemic? What were the associated sentiments?
2. Which subtopics and themes were most salient? With what sentiments were they associated, and how emotional was the discourse?
3. How did the attention to subtopics and themes change over time? How did associated sentiments evolve, and did the discourse become more emotional over time?
4. How was this related to different phases of the pandemic and policy events?

Gaining insights into these RQs adds to the empirical literature on data-driven policymaking. Moreover, this study further advances the methodological literature on extracting, filtering, and analyzing Twitter data for monitoring public debates using web discourse data.

## Methods

### Dataset and Preprocessing

We examined tweets within the time span of January 1, 2020, to January 31, 2022, using the TweetsKB [22] pipeline. TweetsKB is a large-scale knowledge base of annotated tweets harvested using the Twitter streaming application programming interface (API). From 2013 to the transformation of Twitter to X and accompanying limitations regarding API use in May 2024, a random 1% sample of the Twitter stream has been harvested. Their metadata and information automatically extracted from the tweets, such as entities, sentiments, hashtags, and user mentions, are accessible in Resource Description Framework format. The data from January 2013 to December 2020 are available online [23]. We used the same pipeline to harvest tweets for the aforementioned period, resulting in 12,297,163 tweets.

For our study, we analyzed the textual content of tweets, ignoring pictures, links to videos, and other content.

### Relevance Filtering

We extracted relevant tweets by filtering for time (January 1, 2020, to January 31, 2022) using Twitter’s time stamps, language (German) using Twitter’s language tags, and topic (vaccinations). Commonly, researchers rely on manually created seed lists for hashtags or search strings to identify topically relevant tweets [24-30].

However, manual seed lists exhibit a high variance with unknown effects on generated results (eg. the reader may compare the keyword lists used in the cited works to one another. Each study uses a very different keyword list to filter COVID-19–related tweets in the studies by Bonnevie et al [25], Buntain et al [26], and Herrera-Peco et al [27]). They may focus on certain topics or frames while neglecting others; inadequately capture emerging new terms; and suffer from vocabulary mismatch problems, which makes them prone to biases. In addition, their creation may be costly. Therefore, we followed an automatic query term expansion approach to generate a list of search terms (*seed list*).

Starting with an initial query keyword (*Impfung*; German for “vaccination”), we extracted all tweets that contained this keyword as a single token word while not applying case sensitivity. We created a set of candidate terms from this set of tweets by collecting and lemmatizing all verbs, adjectives, nouns, and proper nouns using the spaCy (Explosion AI) part-of-speech tagger [31]. Next, we determined the semantic similarity of each candidate term to the query keyword computing the cosine similarities of their embeddings. We used pretrained word embeddings from FastText (Meta Platforms, Inc) trained on Wikipedia and Common Crawl [32]; we used the German dataset with 300 dimensions [33]. The similarities ranged between −1 and 1. We empirically set the similarity threshold to 0.6 through visual inspection of the resulting candidate keywords and removed all candidates below the threshold. The extracted candidate terms were then ranked by the number of their co-occurrences with the query keyword. The top 30 terms were selected as our seed list (Table S1 in Multimedia Appendix 1). This procedure suggests including terms referring to viruses other than SARS-CoV-2 (eg, the swine influenza). As we assumed such discourses to relate to discourse about COVID-19 in the selected time frame, we did not exclude these keywords from our list. To construct our final set of tweets about COVID-19 vaccinations, we extracted all tweets mentioning at least one of the keywords in their texts or hashtags from the tweets harvested by our TweetsKB pipeline in the specified time frame. This resulted in a set of 201,705 tweets. Removing all tweets written in a language other than German resulted in a set of 199,207 tweets. We adjusted the seed list because the term *Infektion* (German for “infection”) likely added noise as tweets mentioning it alone may not relate to vaccinations. Of the initial 199,207, we excluded 7457 (3.74%) such tweets, leaving a final dataset of 191,750 (96.26%) tweets.

### Sentiment Analysis

We used the automatic tool SentiStrength to identify tweet sentiments. It is tailored for the analysis of short social media texts [34] and measures the strength of both positive and negative sentiments in a tweet on a scale from 1 to 5. Every tweet has 1 score specifying the intensity of the negative sentiment and 1 score specifying the intensity of the positive sentiment. On the basis of the automatically assigned sentiment scores and the tweets’ time stamps, we generated time-series data accumulating all sentiments for 1 day using four different approaches: (1) summing all positive and negative sentiment scores per day, (2) normalizing the summed score by the number of tweets for a relative sentiment score, and (3) counting the number of positive and (4) negative tweets for each day. A tweet is considered positive when the intensity of its positive sentiment is higher than the intensity of its negative sentiment, and vice versa. It should be noted that, for generating the plots, we translated the scores for positive and negative sentiments to intervals of 0 to 4 and −4 to 0, respectively. Using the sum and normalization metrics accounted for intensities of sentiments. For positive and negative sentiments, intensities are translated to positive, negative, and neutral or mixed labels without any information on intensity. All metrics except normalization represented the frequency of tweets in addition to the sentiments. By summing intensity scores, we did not differentiate between tweets that had a neutral sentiment (neither a negative nor a positive sentiment) and tweets with a mixed sentiment (negative and positive sentiments were equally strong).

### Topic Modeling

We used BERTopic [35], a recent transformer-based topic modeling technique, to derive topics from the tweet texts in an unsupervised manner without relying on any previous knowledge. BERTopic allows for the use of custom embeddings. We used the Paraphrase Multilingual MiniLM L12 V2 model [36,37]. Using these multilingual embeddings allowed us to find similarities in sentences within one language or across languages, a valuable property for analyzing German-language tweets that may use English-language terms or quote English-language content. We used BERTopic’s default algorithms, Uniform Manifold Approximation and Projection [38] for dimensionality reduction and Hierarchical Density-Based Spatial Clustering of Applications With Noise [39] for clustering. We computed topics for the complete set of tweets and then classified them into negative and positive. Each tweet was assigned to precisely 1 topic, with 1 noisy residual category for all tweets that did not fit into any of the topic clusters with high probability.

We kept the standard value of 10 documents for the minimum topic size and set the number of topics to 150 to enable a fine-grained analysis while maintaining a topic number that was feasible to review manually. We performed a manual merging step later on by grouping topics into themes. Therefore, we preferred a high number of topics at this step to capture nuance. We manually assigned labels to each topic by interpreting the automatically extracted representative terms (n-gram range set to 1.2; diversity set to 1.0) and by examining the tweets in their corresponding clusters. For this, the first 2 authors of this paper (a computer scientist and a social scientist) labeled all clusters independently and discussed their results.

For some topics, the labels diverged regarding their precise wording but not regarding the perceived content. Final labels were assigned by both authors jointly. For 11 topics, we failed to find suitable labels as the tweets seemed too heterogeneous. We excluded these clusters from our analysis.

### Phases of the Pandemic and Policy Events

We referred to the phases identified by the Robert Koch Institute (RKI) [40], the German government’s central scientific biomedicine institution, to relate the evolution of the discourse to different pandemic phases.

To compare vaccination uptake in the DACH region for each of the different phases and relate it to the evolution in the United Kingdom as an example for other European countries, we added vaccination ratios provided by Our World in Data [41] for an overview of the vaccination uptake in the DACH region in contrast to another European country. Even though the RKI classification refers to the spread of the virus in Germany, Desson et al [6] showed that the German-speaking countries faced similar epidemiological situations during the pandemic. We further investigated the evolution of the discourse in relation to policy events. For this purpose, we identified vaccination policies in the countries within the DACH region, such as the licensing of new vaccines, by drawing on official websites [42-44] and Wikipedia. We derived *policy phases* by grouping similar events.

### Detection of Trends, Peaks, and Change Points

We used the original Mann-Kendall test [45,46] supplied by the *pyMannKendall* package [47] to determine whether there were significant trends in the data regarding sentiments and tweet frequencies. This nonparametric test does not consider serial correlation or seasonal effects. The standard α significance level was set at .05.

For detecting points in which the tweet frequencies peaked or changed in another way in the time-series data, arguably due to shifts in public attention, we computed peaks and change points.

We defined a peak as a point in time where the value deviated from the expected interval (mean and SD) by >1.5 times the expected maximum or minimum value:

([Mean + SD] + [|(mean + SD)| × 1.5])>peak<([mean – SD] – [|(mean – SD)| × 1.5|])

To detect change points, we used the Python library *ruptures* (Python Software Foundation) [48]. We opted for the Prune Exact Linear Time (penalized change point detection) search algorithm, which does not require setting a fixed number of change points in advance. This implementation computes the segmentation, which minimizes the constrained sum of approximation errors for a given model and penalty level [49]. We used the *ruptures* standard parameters.

### Ethical Considerations

The Twitter posts in our dataset were publicly accessible at the time of data collection. We filtered tweets on a per-tweet basis and only retained time stamp, language tag, text, and tweet ID; no user-level metadata or links between tweets and their authors were accessed. Our analysis does not contain any identifying information, and the study is purely observational. Consequently, the study did not meet the criteria for human participant research and did not require a review by an institutional review board.

## Results

### RQ 1: Evolution of Vaccination Discourse in the DACH Region

**Figure 1:**
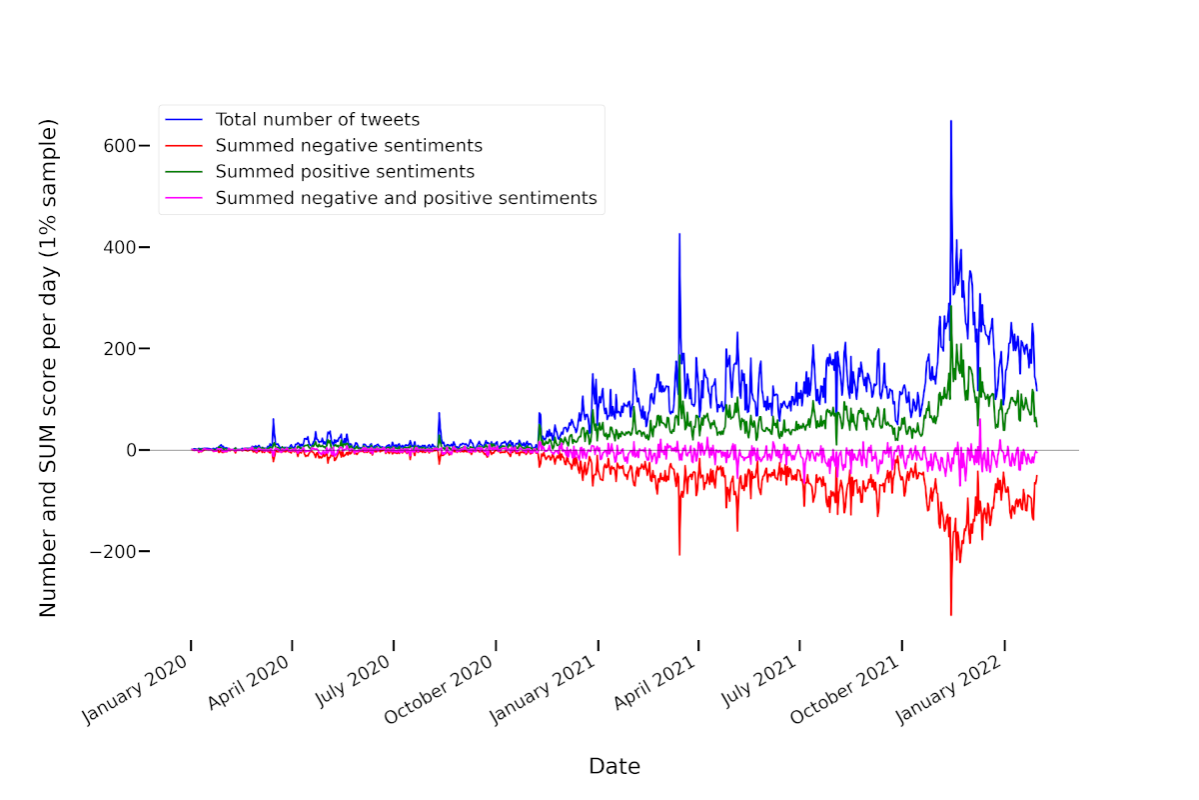
Sentiments and tweet frequencies summed per day to show the evolution of frequencies and sentiment intensities of vaccination-related tweets over time. The black horizontal line marks 0. SUM: sum of all positive and negative sentiment scores.

**Figure 2:**
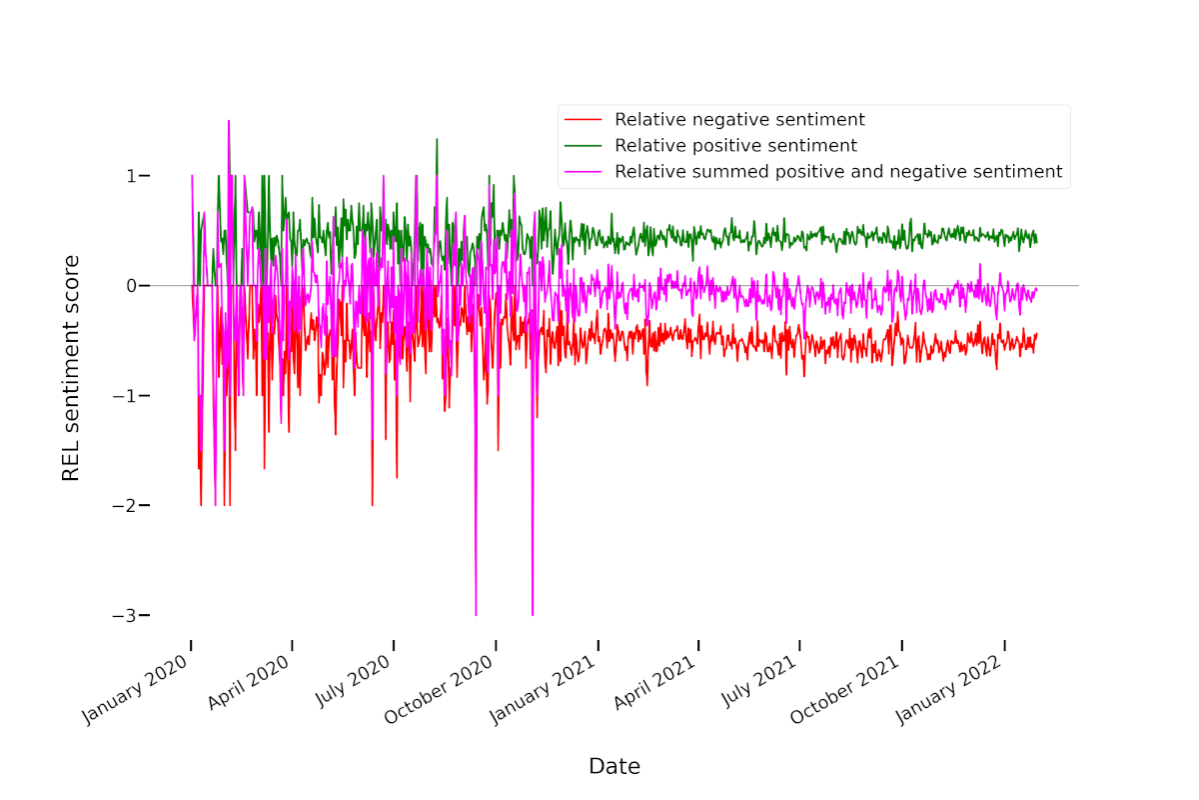
Sentiment intensities summed per day and normalized by the number of tweets (REL) to show the evolution of sentiments in vaccination-related tweets. The black horizontal line marks 0.

As Figure 1 illustrates, before December 2020, only very few tweets mentioned any vaccination-related terms. This affirms that the vaccination discourse captured by our automatically generated seed list was indeed driven by COVID-19 vaccinations. Figure 2 plots the relative sentiment scores (ie, they were normalized with regard to the number of tweets).

Both figures reveal that the overall vaccination discourse showed stronger negative than positive sentiments. Moreover, the discourse became slightly more negative over time. Thus, the negative sentiments were more negative than the positive sentiments were positive. Both the plotted sentiment and tweet frequencies indicate strong fluctuations over time. For the trend analysis using the Mann-Kendall test, we only regarded the time after December 1, 2020, because there was a low number of tweets before this date. We found a positive trend for the positive sentiment intensities (*P*=.007) together with negative trends for negative (*P*<.001) and overall sentiment (*P*=.026). Thus, during this time frame, the discourse became more emotional in the sense that both negative and positive sentiment intensities increased. However, while the summed sentiment intensities were more negative than positive (Figure 1), the number of predominantly positive tweets was slightly higher than the number of predominantly negative tweets—26.73% (51,261/191,750) positive tweets and 24.59% (47,159/191,750) negative tweets (including tweets with neutral or mixed sentiment) over the entire time span. Thus, negative sentiments seemed to be expressed with higher intensity than positive sentiments. However, with means of −0.08 both for the entire time frame (SD 0.33) and for the time after December 1, 2020 (SD 0.11), the average relative sentiment was close to neutral or mixed.

The number of positive and negative tweets, as well as the overall tweet frequency, increased significantly over time (*P*<.001).

**Figure 3:**
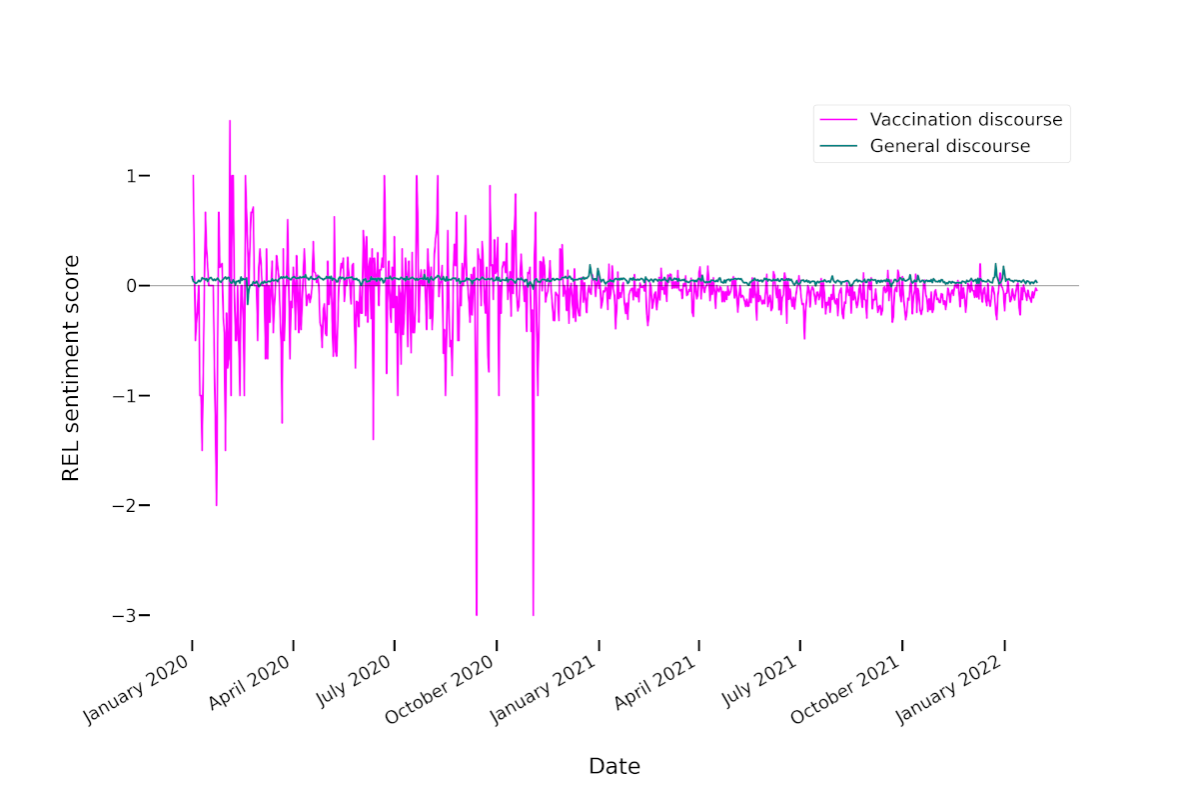
Sentiment intensities summed per day and normalized by the number of tweets (REL) to show the evolution of sentiments in vaccination-related tweets versus the general discourse. To capture the vaccination discourse, these tweets were additionally filtered to contain any vaccination-related terms. The black horizontal line marks 0.

We further investigated whether the negative sentiments were inherent to the vaccination discourse or due to an overall negative German-language Twitter discourse. For that, we analyzed the sentiments for all German-language tweets harvested using our pipeline during the investigated time frame. Figure 3 relates the sentiments in the German-language vaccination tweets to the sentiments in German-language tweets of all topics in the same time frame. The depicted sentiments are the normalization metric scores.

The strong fluctuations in sentiment at the beginning of the year 2020 for the vaccination-related tweets can be attributed to the relatively low number of tweets in that time frame (Figure 1). Similarly, the vaccination sentiments seemed to exhibit higher fluctuations due to the smaller number of tweets compared to the general Twitter discourse.

The results show that the discourse on vaccinations was more negative than the general discourse in German-language tweets. In the latter, the sentiment was overall more positive than negative both in terms of summed and relative sentiment intensities, with a mean of 0.05 (SD 0.02) for the normalization metric score (as compared to −0.08 for the vaccination tweets), and in terms of numbers of tweets, with 14.3% (1,758,776/12,297,163) negative and 24.53% (3,015,915/12,297,163) positive tweets. There was also a significant negative trend for the general German-language Twitter discourse, which was caused by both the negative sentiments becoming more negative (*P*<.001) and the positive sentiments becoming less positive (*P*<.001) according to the Mann-Kendall trend analysis. Numbers for negative, positive, and all tweets showed a significant increasing trend (*P*<.001). While the summed positive and negative sentiments were relatively close to balancing each other out for the vaccination-related discourse, the increasing trend in positive sentiment scores and the decreasing trend in negative sentiment scores suggest that the discourse was indeed rather emotional and increasingly so. Both the tweet frequencies and sentiments also fluctuated heavily at different points in time. The following sections investigate which topics were discussed in general and when tweet frequencies increased (ie, which topics were the focus of attention and which topics were responsible for positive and negative sentiments and sentiment trends).

### RQ 2: Topics, Sentiments, and Themes

#### Topics and Associated Sentiments

The vaccination discourse covered a wide range of topics (refer to Table 1 for the top 30).

The most frequently discussed topic addressed the question of whether *children* should be vaccinated, how they could be protected, their role in transmitting the virus, and their role in the pandemic more generally.

**Table 1:** The 30 most frequent vaccination-related topics. Observational data extracted from a 1% sample of tweets filtered for time stamps ranging from January 1, 2020, to January 31, 2022; German-language tags; and tweet texts containing any vaccination-related terms; grouped by topic^a^.

Rank	Topic label	Tweets, n (%)	Mean sentiment per tweet (SD)
1	Children	8892, 191750 (4.64%)	−0.12 (1.18)
2	Anecdotes: Experience with Corona vaccination	2210, 191750 (1.15%)	−0.17 (1.29)
3	(Prominent) vaccinated and unvaccinated men	2058, 191750 (1.07%)	−0.07 (1.05)
4	Situation in Germany and comparisons	1796, 191750 (0.94%)	−0.07 (1.03)
5	“Do (not) get vaccinated”	1738, 191750 (0.91%)	0.16 (0.90)
6	COVID-19 and other flu viruses, severity of the virus	1600, 191750 (0.83%)	−0.25 (1.16)
7	COVID-19 in Israel	1533, 191750 (0.80%)	−0.20 (1.16)
8	Regulations (eg, access restrictions for unvaccinated persons)	1476, 191750 (0.77%)	−0.04 (1.05)
9	“AstraZeneca vaccine”	1425, 191750 (0.74%)	−0.01 (1.04)
10	Duration of vaccination protection	1382, 191750 (0.72%)	0.04 (1.00)
11	Lockdowns	1132, 191750 (0.59%)	−0.10 (1.08)
12	Mutations and virus spread when vaccinated	1118, 191750 (0.58%)	−0.11 (1.09)
13	Basic rights	1093, 191750 (0.57%)	0.14 (1.03)
14	Practical implementation, opportunities to get vaccinated	1055, 191750 (0.55%)	0.39 (0.85)
15	Propaganda and fake news, also at the meta level	1021, 191750 (0.53%)	−0.34 (1.10)
16	Vaccinations for elderly people	988, 191750 (0.5%)	−0.13 (1.08)
17	Compulsory vaccination	938, 191750 (0.5%)	−0.15 (1.12)
18	Meta-discussion about Twitter vaccination discourse	761, 191750 (0.4%)	−0.15 (1.22)
19	Masks and mask mandate	729, 191750 (0.4%)	−0.15 (1.17)
20	Vaccine efficacy for Omicron	676, 191750 (0.4%)	−0.17 (1.10)
21	Compulsory vaccination at work	651, 191750 (0.3%)	−0.06 (1.06)
22	SARSCoV2 (tweets using the SARSCoV2 term or hashtag)	635, 191750 (0.3%)	−0.12 (1.25)
23	Demonstrations and protests	633, 191750 (0.3%)	−0.04 (0.98)
24	Merkel (Germany’s chancellor at the time)	625, 191750 (0.3%)	−0.07 (1.09)
25	Vaccinations for children: medical views	611, 191750 (0.3%)	−0.08 (1.05)
26	mRNA vaccines	567, 191750 (0.3%)	−0.02 (0.96)
27	Immune system	550, 191750 (0.3%)	−0.19 (1.14)
28	Statistics about vaccination uptake and policies	535, 191750 (0.3%)	−0.08 (0.92)
29	#AllesInDenArm	476, 191750 (0.2%)	−0.01 (1.08)
30	Austria	472, 191750 (0.2%)	0.00 (0.93)

^a^Topics were extracted using BERTopic on the tweet texts, and the labels are assigned manually by the first 2 authors. Topics are ranked by the number of tweets assigned to them to list the top 30 vaccination-related topics. Sentiments were extracted using the SentiStrength tool, summed, and normalized by their number.

Some topics referred to similar issues with varying levels of granularity, for example, the duty to vaccinate in general (rank 17) versus the duty to vaccinate at work (rank 21) and general calls to action (rank 5) versus tweets of the #AllesInDenArm (everythingIntoTheArm) campaign (rank 29), in which prominent individuals and ordinary Twitter users posted a specific hashtag to communicate their vaccination status and motivate others to get vaccinated.

This restricted the informative value of the frequency rankings. Thus, we manually identified more general relevant themes to investigate their salience over time and their relationships to vaccination policy events.

#### Themes

We adopted the same workflow as for generating topic labels. Each of the first 2 authors examined all topic labels and mapped them to themes. On this basis, we arrived at the following final set of themes that included at least 3 topics (Table S2 in Multimedia Appendix 1): (1) freedom and civil liberties, (2) safety and side effects of vaccinations, (3) effectiveness of vaccinations, (4) mobilization, (5) details about the vaccination campaign, (6) conspiracy theories, (7) country comparisons, (8) influential individuals and their stances or behaviors, (9) specific vaccines, and (10) data about the pandemic. This analysis revealed that, while many tweets concerned health-related issues (*safety and side effects of vaccinations*, *effectiveness of vaccinations*, and *specific vaccines*), a very high number of tweets focused on the effects of policies on society.

Overall, Twitter users seemed to be similarly concerned about freedom and civil liberties and health-related issues. It should be noted that the theme *conspiracy theories* contained tweets discussing different theories (eg, concerning Bill Gates’ motives regarding vaccinations) but also sarcastic tweets and tweets discussing news coverage and perceived propaganda at a meta level. Therefore, many tweets assigned to this theme cannot be interpreted as a high level of belief in conspiracies or media distrust. Instead, this signals a high attention to these topics.

### RQ 3: Topic and Theme Sentiments Over Time

#### Topic Sentiments: Complete Time Interval

The average sentiment scores support the analysis in the Evolution of Vaccination Discourse in the DACH Region section. The sentiments for many topics were neither very negative nor very positive when averaged over the entire time span, with a few exceptions (eg, *practical implementation* [0.39], which encompassed many tweets with people celebrating their vaccination appointments; *propaganda and fake news* [−0.34]; and *COVID-19 and other flu viruses* [−0.25]).

While sentiments are not to be interpreted as stances (ie, negative sentiments do not necessarily signal disapproval), negative scores suggest that these topics were coupled with a focus on negative aspects in the discussion.

For the Mann-Kendall test, we considered the relative sentiment scores starting on December 1, 2020, ignoring the low number of tweets before that time (Figure 2). The Mann-Kendall test revealed significant negative trends regarding the relative sentiment for the following 13% (4/30) of the topics: *anecdotes: experience with COVID-19 vaccination* (*P*=.013), *COVID-19 in Israel* (*P*<.001), *propaganda and fake news* (*P*=.009), and *vaccine efficacy for Omicron* (*P*<.001).

In total, 3 topics showed a positive trend: *COVID-19 and other flu viruses* (*P*=.017), *basic rights* (*P*<.001), and *practical implementation* (*P*=.001). No significant trends were found for the remaining topics (c*hildren* (*P*=.2), *[prominent] vaccinated and unvaccinated men* (*P*=.2), *situation in Germany and comparisons* (*P*=.3), *“do (not) get vaccinated”* (*P*>.9), *regulations* (*P*=.8), *AstraZeneca vaccine* (*P*=.3)*, duration of vaccination protection* (*P*=.1)*, lockdowns* (*P*=.2)*, mutations and virus spread when vaccinated* (*P*=.7)*, vaccinations for elderly people* (*P*=.2)*, compulsory vaccination* (*P*=.3)*, meta-discussion about Twitter vaccination discourse* (*P*=.1)*, masks and mask mandate* (*P*=.7)*, compulsory vaccination at work* (*P*=.7)*, SARSCoV2* (*P*=.05)*, demonstrations and protests* (*P*=.2)*, Merkel* (*P*=.2)*, vaccinations for children: medical views* (*P*=.5)*, mRNA vaccines (P*=*.1), immune system* (*P*=.08)*, statistics about vaccination uptake and policies* (*P*=.8)*, #AllesInDenArm* (*P*=.4)*,* Austria (*P*=.9).

When analyzing the relative positive and negative sentiment scores of all tweets separately, we observed that, for 56.67% (17/30) of the topics, the negative intensities became significantly more negative, whereas the positive intensities became significantly more positive (*propaganda and fake news* (*P*<.001, *P*<.001), *compulsory vaccination* (*P*=.002, *P*<.001), *meta-discussion about Twitter vaccination discourse* (*P*<.001, *P*<.001), *masks and mask mandates* (*P*<.001, *P*<.001), *vaccine efficacy for Omicron* (*P*<.001, *P*<.001), *compulsory vaccination at work* (*P*<.001, *P*<.001), *demonstrations and protests* (*P*<.001, *P*<.001), *vaccinations for children: medical views* (*P*<.001, *P*<.001), *immune system* (*P*<.001, *P*<.001), *#AllesInDenArm* (*P*<.001, *P*<.001), *Austria* (*P*=.016, *P*=.002), *anecdotes: experience with COVID-19 vaccination* (*P*<.001, *P*<.001), *[prominent] vaccinated and unvaccinated men* (*P*<.001, *P*=.014), *regulations* (*P*<.001, *P*<.001), *duration of vaccination protection* (*P*<.001, *P*<.001), *mutations and virus spread when vaccinated* (*P*=.017, *P*=.015), and *practical implementation* (*P*<.047, *P*<.001).

Only for 7% (2/30) of the topics (*Merkel* and *AstraZeneca vaccine*) we found the opposite—the positive sentiments became less positive, and the negative sentiments became less negative over time (*P*<.001).

These findings suggest that the discourse overall became more emotional over time. In later sections, we check whether this also holds true for the vaccination discourse beyond the most prominent individual topics and analyze the shifts in public attention in more detail.

#### Theme Sentiments: Complete Time Interval

Again analyzing the time frame starting on December 1, 2020, we found 2 themes with a positive trend in the overall relative sentiment: *details of the vaccination campaign* (*P*<.001) and *specific vaccines* (*P*=.025). For *details of the vaccination campaign*, the positive sentiments became significantly more positive (*P*=.004), and the negative sentiments became significantly less negative (*P*=.008).

For *specific vaccines*, the negative sentiments became less negative (*P*<.001), with no significant change for the positive sentiments (*P*=.06). In total, 3 themes had a significant negative overall trend—*influential individuals* (*P*<.001), *country comparisons* (*P*=.007), and *conspiracy theories* (*P*=.009)—caused by the negative sentiments becoming increasingly negative (*P*<.001, *P*=.007, *P*<.001), with no trends concerning the positive sentiments for *influential individuals* (*P*=.3) and *country comparisons* (*P*=.7)*,* and a weaker positive trend for the positive sentiments for *conspiracy theories* (*P*=.048).

The themes *effectiveness of vaccinations* and *freedom and civil liberties* showed no significant trends (*P*=.9, *P*=.2, *P*=0.1 for *effectiveness of vaccinations* and *P*=.9, *P*=.9, *P*=.9 for *freedom and civil liberties*, overall, concerning negative sentiments, and concerning positive sentiments, respectively); *mobilization* and *safety and side effects* showed significant negative trends in negative sentiment intensities (*P*=.001, *P*=.006). Changes in positive sentiment intensities (*P*>.9, *P*=.07) and overall sentiment (*P*=.1, *P*=.2) were not significant.

We analyze the evolution of tweet frequencies for the themes in more detail in the following section.

### RQ 4: Relationship to Pandemic Phases and Policy Events

#### Pandemic Phases and Policy Events

The phases from the beginning of the pandemic until the end of the time under investigation in this study were classified as listed in Table 2.

We identified 57 policy events: 22 (39%) for Germany, 16 (28%) for Switzerland, and 19 (33%) for Austria (Tables S3-S5 in Multimedia Appendix 1). The 3 countries partly issued similar policies at similar times, which did not fully coincide with the pandemic phases (Table 3).

**Table 2:** Phases of the pandemic as classified by the Robert Koch Institute (own translation of the phase labels; calendar weeks mapped to dates) with added vaccination ratio statistics provided by Our World in Data. Averaged values for the Deutschland (Germany), Austria, and CH (Confoederatio Helvetica, Latin for Switzerland) (DACH) region have been added by the authors of this paper).

Pandemic phase	Beginning	End	Vaccinated people in Germany, %	Vaccinated people in Austria, %	Vaccinated people in Switzerland, %	Vaccinated people in the DACH region (%), mean (SD)	Vaccinated people in the United Kingdom, %
Sporadic cases	January 27, 2020	March 2, 2020	—^a^	—	—	—	—
Wave 1	March 2, 2020	May 18, 2020	—	—	—	—	—
Summer 2020 plateau	May 18, 2020	September 28, 2020	—	—	—	—	—
Wave 2	September 28, 2020	March 1, 2021	5.2	5.2	6.4	5.6 (0.57)	30
Wave 3	March 1, 2021	June 14, 2021	49	48	44	47 (2.16)	62
Summer 2021 plateau	June 14, 2021	August 2, 2021	63	60	55	59 (3.30)	70
Wave 4	August 2, 2021	December 27, 2021	75	74	69	73 (2.62)	77
Wave 5	December 27, 2021	January 31, 2022^b^	77	76	70	74 (3.09)	78

^a^Not applicable.

^b^End of the investigated time frame.

**Table 3:** Policy phases for Germany, Austria, and Switzerland derived by manually grouping 57 COVID-19 vaccination–related policy events as mentioned on official websites for the time frame from January 1, 2020, to January 31, 2022.

Policy phase	Beginning	End	Description
I	November 1, 2020	December 10, 2020	Beginning of the official COVID-19 vaccination policies
II	December 10, 2020	April 15, 2021	Publishing of vaccination strategies; authorization of the vaccines and vaccinations start; halt, and resumption of AstraZeneca vaccinations in Germany
III	April 15, 2021	May 15, 2021	Suspension of priority groups for AstraZeneca vaccines in Germany; international vaccination certificate preparations in Switzerland
IV	May 15, 2021	November 1, 2021	Vaccine recommendations for specific age groups; access restrictions for unvaccinated persons in Germany
V	November 1, 2021	January 31, 2022	Booster shot recommendations and authorizations of vaccines for children; AstraZeneca vaccination stop in Germany; lockdowns for unvaccinated people under certain conditions in Austria

#### Relationship Between Themes and Pandemic Phases

Table 4 lists the frequencies and connected sentiments of all themes during different phases of the pandemic. As the phases differ in duration and the number of tweets increased over time, we also list the relative number of tweets as a percentage of the tweets categorized under each of the themes. We excluded the *sporadic cases* phase from the following analysis as it did not contain enough tweets to derive meaningful rankings.

**Figure 4:**
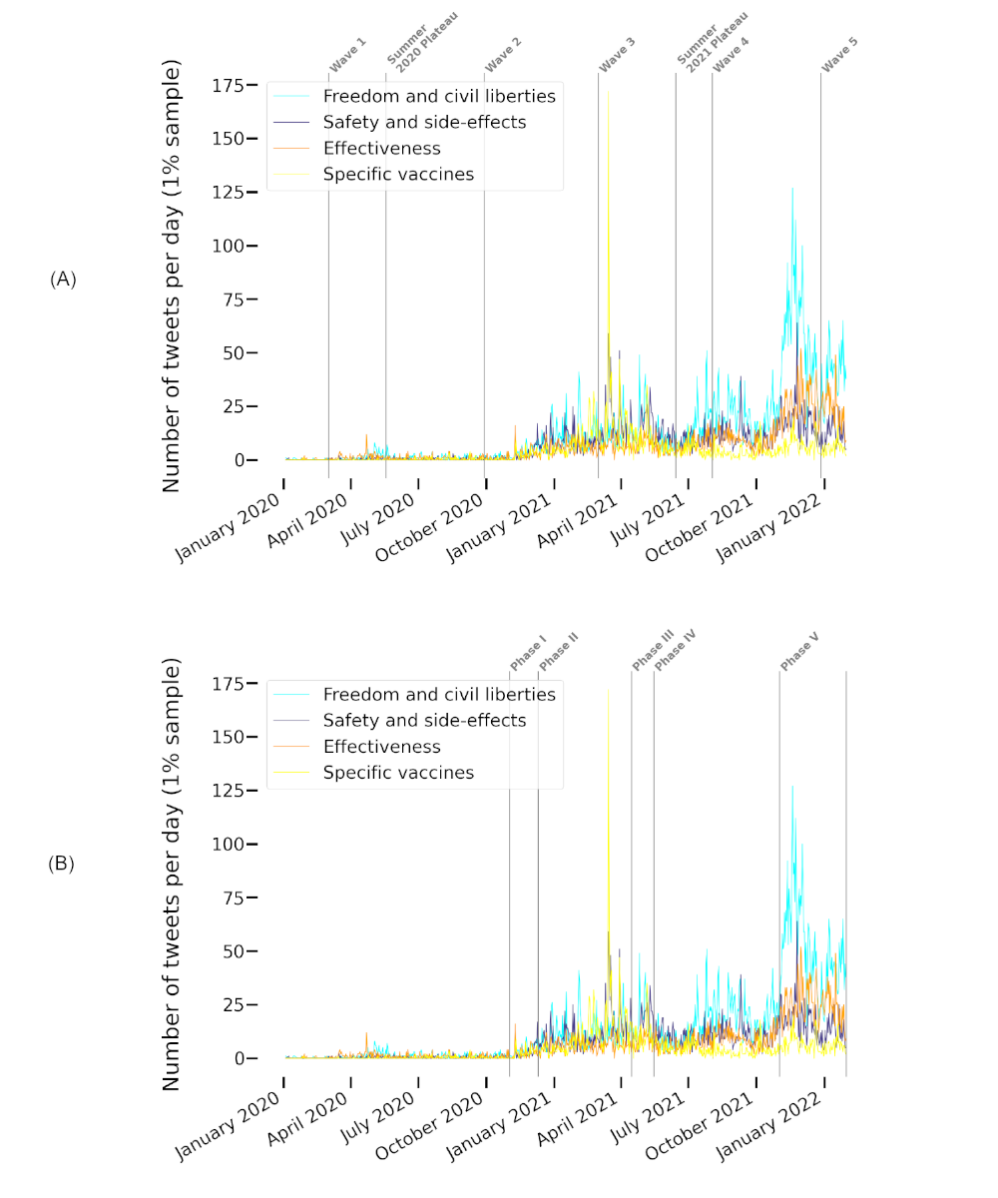
The evolution of daily tweet frequencies of the themes freedom and civil liberties, safety and side effects, effectiveness of vaccinations, and specific vaccines related to different (A) pandemic and (B) policy phases as classified by the Robert Koch Institute and by the authors of this paper, respectively. Gray vertical lines mark the beginning of a phase.

The themes *freedom and civil liberties* and *country comparisons* were prominent throughout all phases of the pandemic—they had the highest (ie, the top) mean ranks across all time intervals (1.71 and 2.86, respectively) and a high rank stability with SDs of 1.11 and 1.35, respectively. For *freedom and civil liberties*, we observed an increased frequency, both absolute and relative, in the last 2 phases (ie, it received more attention during the later phases than during the early ones). Both themes had their lowest rank during the first wave and their second-lowest rank during wave 3. The top 3 theme *effectiveness of vaccinations* (mean rank 4.14) also received the least attention in wave 3. This phase was dominated by the *safety and side effects* theme (mean rank 5.29). Ranking third in wave 3 behind *freedom and civil liberties*, we found *specific vaccines*. In other phases, *specific vaccines* also received attention but to a lesser degree (mean rank 6.86). *Safety and side effects*, *specific vaccines*, and *effectiveness of vaccinations* showed the greatest fluctuations in ranks, with SDs of 2.43, 2.27, and 2.12, respectively. Only *conspiracy theories* fluctuated more (SD 3.21). The development of the highly fluctuating themes s*afety and side effects*, *effectiveness of vaccinations*, and s*pecific vaccines* and the dominating theme *freedom and civil liberties* is illustrated in Figure 4.

This analysis shows that attention to topics in wave 3 differed from that in the other phases and that directly vaccine-related themes were the most unstable. We investigate possible reasons in more detail in the next section.

**Table 4:** Tweet frequencies and sentiments by theme and pandemic phase. Observational data extracted from a 1% sample of tweets filtered for time stamps ranging from January 1, 2020, to January 31, 2022; German-language tags; and tweet texts containing any vaccination-related terms; grouped by pandemic phases and themes.a

Rank and topic				Tweets, n (%)		Mean sentiment per tweet, (SD)
**Sporadic cases**						
	1	Country comparisons	16, 47 (34.04)		0.19 (1.33)	
	2	Effectiveness of vaccinations	6, 47 (12.77)		0.33 (0.75)	
	3	Conspiracy theories	5, 47 (10.64)		−0.20 (0.75)	
	3	Mobilization	5, 47 (10.64)		0.20 (1.17)	
	4	Influential individuals and their stances or behaviors	4, 47 (8.51)		0.75 (0.43)	
	5	Details of the vaccination campaign	3, 47 (6.38)		−1.33 (1.25)	
	5	Data about the pandemic	3, 47 (6.38)		0.00 (0.0)	
	5	Freedom and civil liberties	3, 47 (6.38)		0.00 (1.63)	
	6	Safety and side effects	1, 47 (2.13)		0.00 (0.0)	
	6	Specific vaccines	1, 47 (2.13)		0.00 (0.0)	
**Wave 1**						
	1	Conspiracy theories	176, 729 (24.14)		0.06 (0.87)	
	2	Influential individuals and their stances or behaviors	154, 729 (21.12)		−0.14 (0.92)	
	3	Effectiveness of vaccinations	104, 729 (14.27)		−0.05 (1.07)	
	4	Freedom and civil liberties	95, 729 (13.03)		−0.01 (1.19)	
	5	Country comparisons	70, 729 (9.6)		0.17 (1.1)	
	6	Mobilization	43, 729 (5.9)		0.19 (0.87)	
	7	Data about the pandemic	39, 729 (5.35)		−0.05 (0.81)	
	8	Safety and side effects	29, 729 (3.98)		0.03 (1.03)	
	9	Details of the vaccination campaign	13, 729 (1.78)		0.31 (0.82)	
	10	Specific vaccines	6, 729 (0.82)		0.33 (0.47)	
**Summer 2020 plateau**						
	1	Country comparisons	202, 836 (24.16)		−0.04 (0.94)	
	2	Freedom and civil liberties	137, 836 (16.39)		0.05 (1.06)	
	3	Effectiveness of vaccinations	104, 836 (12.44)		0.11 (1.09)	
	4	Conspiracy theories	92, 836 (11)		−0.02 (0.93)	
	5	Influential individuals and their stances or behaviors	91, 836 (10.89)		0.09 (1.03)	
	6	Mobilization	62, 836 (7.42)		0.35 (0.93)	
	7	Specific vaccines	50, 836 (5.98)		−0.08 (0.87)	
	8	Safety and side effects	42, 836 (5.02)		−0.17 (1.25)	
	9	Details of the vaccination campaign	30, 836 (3.59)		0.13 (1.02)	
	10	Data about the pandemic	26, 836 (3.11)		0.08 (1.07)	
**Wave 2**						
	1	Freedom and civil liberties	1139, 6595 (17.27)		−0.08 (1.07)	
	2	Country comparisons	1101, 6595 (16.69)		−0.06 (1.06)	
	3	Influential individuals and their stances or behaviors	865, 6595 (13.12)		0.06 (1.04)	
	4	Safety and side effects	812, 6595 (12.31)		−0.18 (1.09)	
	5	Specific vaccines	738, 6595 (11.19)		−0.05 (1.06)	
	6	Effectiveness of vaccinations	610, 6595 (9.25)		−0.10 (1.11)	
	7	Mobilization	532, 6595 (8.07)		0.16 (1.02)	
	8	Details of the vaccination campaign	403, 6595 (6.11)		0.06 (0.96)	
	9	Conspiracy theories	266, 6595 (4.03)		−0.10 (1.01)	
	10	Data about the pandemic	129, 6595 (1.96)		0.06 (0.82)	
**Wave 3**						
	1	Safety and side effects	1611, 9179 (17.55)		−0.14 (1.08)	
	2	Freedom and civil liberties	1390, 9179 (15.14)		−0.04 (1.08)	
	3	Specific vaccines	1384, 9179 (15.08)		−0.01 (1.0)	
	4	Country comparisons	1254, 9179 (13.66)		−0.06 (1.01)	
	5	Mobilization	995, 9179 (10.84)		0.19 (0.90)	
	6	Influential individuals and their stances or behaviors	789, 9179 (8.6)		−0.02 (1.06)	
	7	Effectiveness of vaccinations	711, 9179 (7.75)		−0.09 (1.1)	
	8	Details of the vaccination campaign	687, 9179 (7.48)		0.12 (1.01)	
	9	Conspiracy theories	220, 9179 (2.4)		−0.10 (1.17)	
	10	Data about the pandemic	138, 9179 (1.5)		0.03 (0.82)	
**Summer 2021 plateau**						
	1	Freedom and civil liberties	742, 4100 (18.1)		−0.09 (1.13)	
	2	Country comparisons	675, 4100 (16.46)		−0.26 (1.12)	
	3	Mobilization	569, 4100 (13.88)		0.14 (0.89)	
	4	Details of the vaccination campaign	504, 4100 (12.29)		0.32 (0.88)	
	5	Safety and side effects	487, 4100 (11.88)		−0.24 (1.12)	
	6	Effectiveness of vaccinations	359, 4100 (8.76)		−0.17 (1.1)	
	7	Influential individuals and their stances or behaviors	324, 4100 (7.9)		−0.10 (1.05)	
	8	Specific vaccines	234, 4100 (5.71)		−0.03 (1.0)	
	9	Conspiracy theories	130, 4100 (3.17)		−0.23 (1.23)	
	10	Data about the pandemic	76, 4100 (1.85)		0.17 (0.92)	
**Wave 4**						
	1	Freedom and civil liberties	5140, 18511 (27.77)		−0.07 (1.08)	
	2	Effectiveness of vaccinations	2362, 18511 (12.76)		−0.09 (1.07)	
	3	Country comparisons	2341, 18511 (12.65)		−0.20 (1.11)	
	4	Mobilization	2335, 18511 (12.61)		0.07 (1.0)	
	5	Safety and side effects	2073, 18511 (11.2)		−0.14 (1.12)	
	6	Influential individuals and their stances or behaviors	1756, 18511 (9.49)		−0.16 (1.13)	
	7	Details of the vaccination campaign	958, 18511 (5.18)		0.20 (0.91)	
	8	Specific vaccines	702, 18511 (3.79)		0.13 (0.9)	
	9	Conspiracy theories	543, 18511 (2.93)		−0.27 (1.08)	
	10	Data about the pandemic	301, 18511 (1.63)		0.13 (0.66)	
**Wave 5**						
	1	Freedom and civil liberties	1403, 4865 (28.84)		−0.06 (1.08)	
	2	Effectiveness of vaccinations	831, 4865 (17.08)		−0.09 (1.06)	
	3	Country comparisons	622, 4865 (12.79)		−0.04 (1.0)	
	4	Influential individuals and their stances or behaviors	513, 4865 (10.54)		−0.11 (1.09)	
	5	Mobilization	483, 4865 (9.93)		0.10 (1.0)	
	6	Safety and side effects	428, 4865 (8.8)		−0.30 (1.05)	
	7	Specific vaccines	189, 4865 (3.88)		0.04 (0.9)	
	8	Conspiracy theories	180, 4865 (3.7)		−0.39 (1.05)	
	9	Details of the vaccination campaign	167, 4865 (3.43)		0.46 (0.89)	
	10	Data about the pandemic	49, 4865 (1.01)		−0.02 (0.77)	

^a^Topics were extracted using BERTopic on the tweet texts, and the topics were manually grouped into themes by the first 2 authors. Themes are ranked by the number of tweets assigned to them. Sentiments were extracted with the SentiStrength tool, summed, and normalized by their number. Numbers were computed for each pandemic phase individually to compare the theme frequencies at different phases of the pandemic.

#### Relationship Between Themes and Policy Events

**Figure 5:**
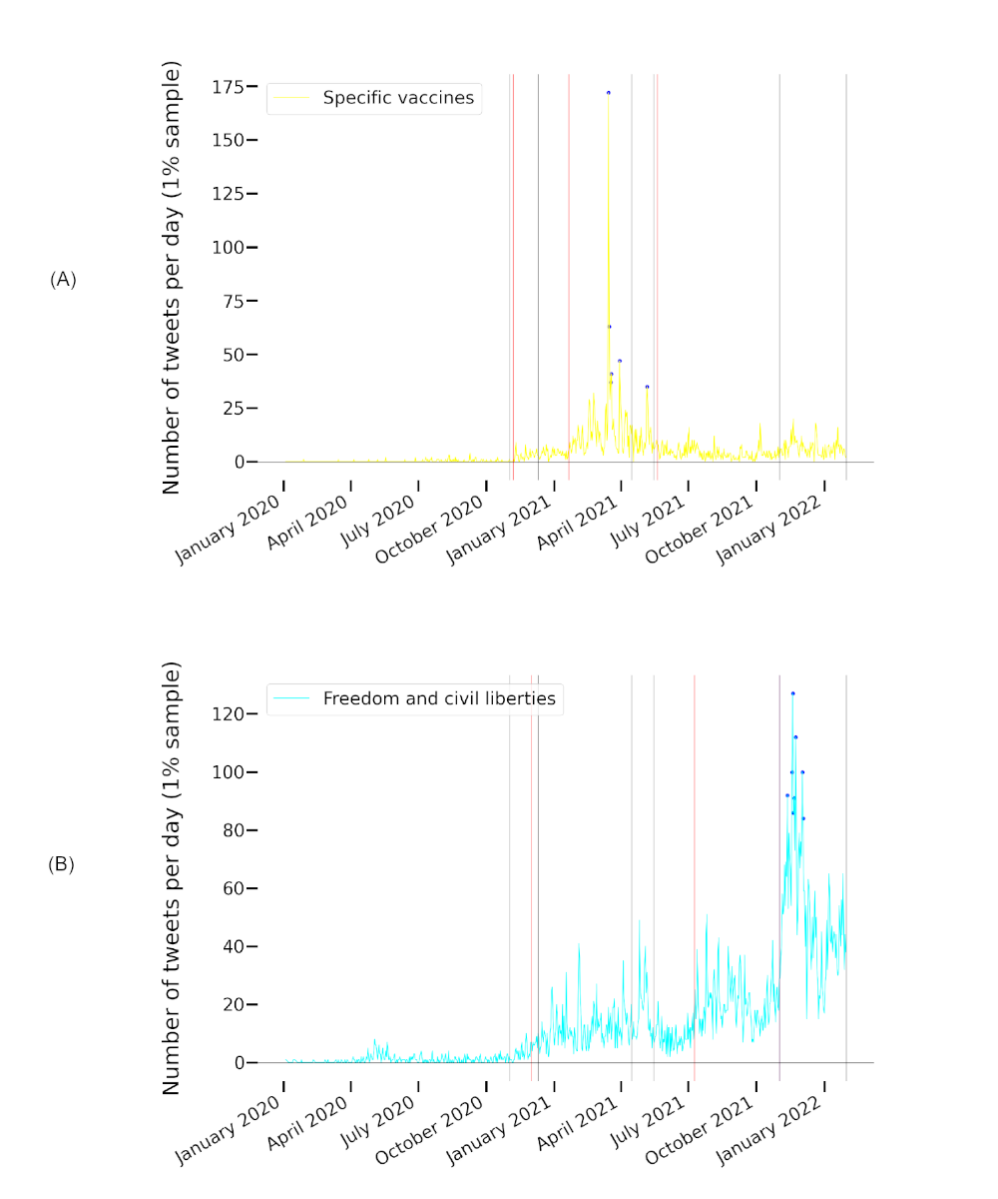
The change points and peaks of daily tweet frequencies of the themes (A) specific vaccines (B) freedom and civil liberties related to different policy phases as classified by the authors of this paper. Change points were detected using the Prune Exact Linear Time algorithm. Peaks designate values that deviate from the expected interval by >1.5 times the expected maximum or minimum value. Gray lines mark the beginning of policy phases, red lines mark the change points, purple lines mark the beginning of policy phases and change points on the same day, and blue dots mark peaks.

**Figure 6:**
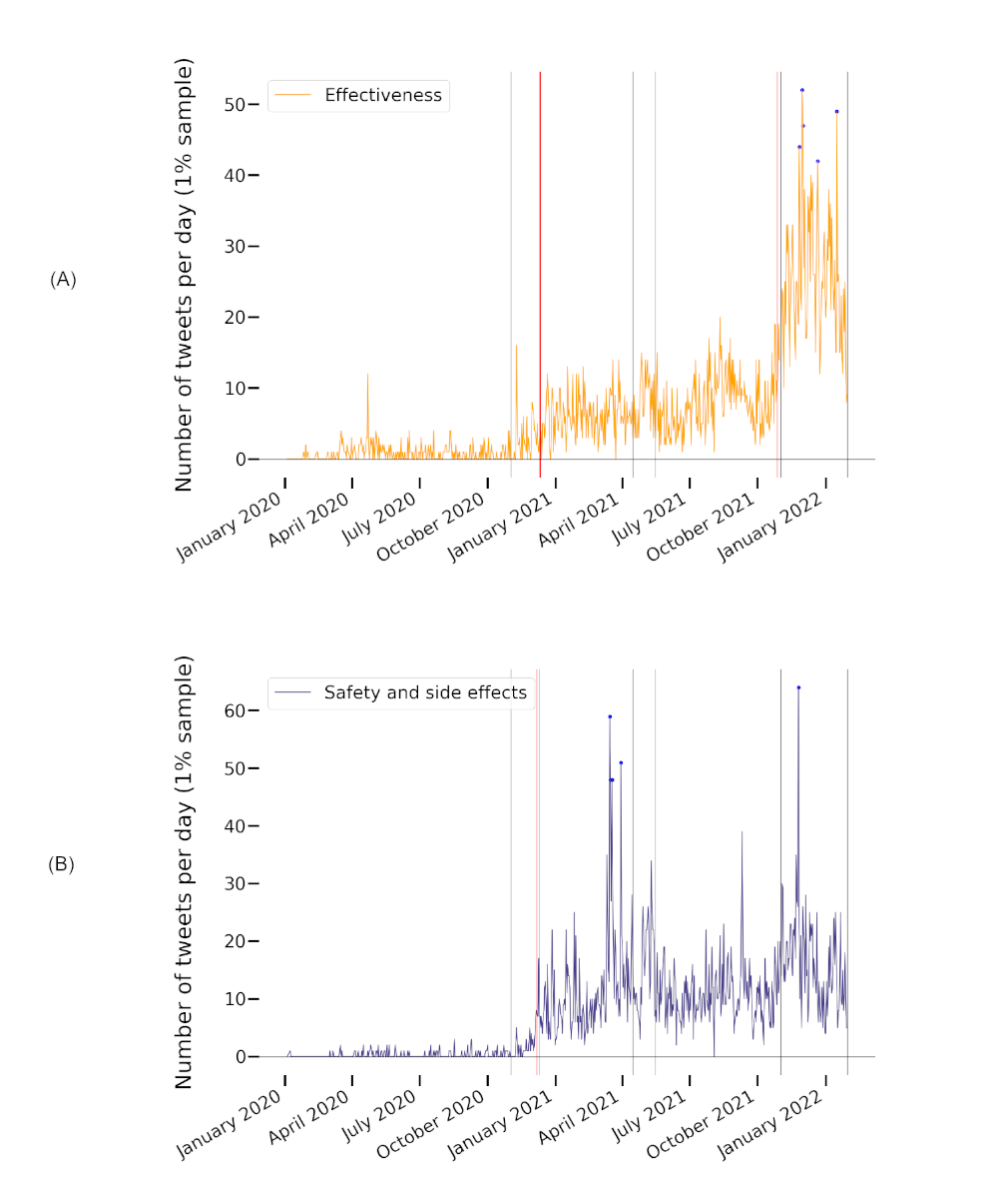
The change points and peaks of daily tweet frequencies of the themes (A) effectiveness of vaccinations and (B) safety and side effects related to different policy phases as classified by the authors of this paper. Change points were detected using the Prune Exact Linear Time algorithm. Peaks designate values that deviate from the expected interval by >1.5 times the expected maximum or minimum value. Gray lines mark the beginning of policy phases, red lines mark the change points, purple lines mark the beginning of policy phases and change points on the same day, and blue dots mark peaks.

To reveal possible connections to policy actions, we investigated the relationship between change points and peaks for the most fluctuating and dominant themes in Figure 4. In Figure 4A, we relate them to the pandemic phases as classified by the RKI, in Figure 4B, we relate them to the policy phases introduced in Table 2. The development of the themes seems to align better with the policy phases than with the pandemic phases as classified by the RKI. This is further supported by the analysis of change points and peaks as visualized in Figures 5 and 6.

Phase I, marking the beginning of official COVID-19 vaccination policies in the DACH region (Table 2), went along with change points in tweet numbers for *specific vaccines*, which started to be discussed frequently after this point. After the preparations for and the actual vaccination rollouts (phase II), the themes *effectiveness of vaccinations*, *safety and side effects*, and *freedom and civil liberties* received increasing attention.

When AstraZeneca vaccinations were halted in Germany due to safety concerns and resumed a few weeks later (Table S3 in Multimedia Appendix 1), we observed peaks for the themes s*afety and side effects* and *specific vaccines* (both themes: March 15, March 16, March 18, and March 30, 2021; for *specific vaccines* additionally on March 19, 2021). On May 6, 2021, during the third phase, vaccinations with the AstraZeneca vaccine were possible for all individuals regardless of priority group membership. A peak for *specific vaccines* can be found on the same day. Policy phase IV involved few policy events and few topic rank fluctuations, change points, and peaks.

Restrictions for unvaccinated persons were discussed and finally implemented in Germany in August 2021, and *freedom and civil liberties* was the dominant theme. November 2021 marked the month of booster recommendations and authorizations of vaccines for children. In addition, there was a nationwide lockdown for unvaccinated persons in Austria (Table S4 in Multimedia Appendix 1). This and the following month contained the all-time peaks of the *freedom and civil liberties* and *effectiveness of vaccinations* themes.

This analysis suggests that the high fluctuation of attention to the themes s*afety and side effects*, *effectiveness of vaccinations*, and *specific vaccines* and specific policy actions might have been related.

## Discussion

### Principal Findings

Our results show that vaccinations were controversially discussed—the total number of tweets about this important societal issue increased over time, and the sentiments in the discourse became both more emotional and more negative (RQ 1). Generally, discourse about COVID-19 vaccinations was significantly more negative than the average discourse on Twitter during the same period. Investigating RQ 2 and RQ 3, we found that the Twitter discourse showed fluctuations in the topics and themes that were at the center of public attention—while medical concerns such as the safety and side effects of vaccinations were prominently discussed early in the debate and concerning specific events, the focus increasingly shifted to a discussion of broader societal concerns, especially those regarding freedom and civil liberties. At the same time, vaccination acceptance and uptake were low early in the pandemic and increased over time. Our investigations into RQ 4 provide insights into possible drivers of these changes—shifts in the discourse aligned better with policy phases than with pandemic phases. Peaks of attention to themes were related to policy events such as halting AstraZeneca vaccinations or incentivizing vaccinated persons.

### Implications and Considerations for Policymaking

This section provides a more detailed discussion of those findings, including interpretations, implications, and comparisons to existing literature.

Overall, we found that, when the COVID-19 vaccines were first authorized, the debate on Twitter focused on a range of topics, including *side effects of individual vaccines* and *vaccinations in general* but also *freedom and civil liberties*. During later phases of the pandemic, when different policies restricting the freedom of unvaccinated citizens were publicly discussed and later implemented, the attention increasingly shifted away from medical and other concerns toward questions of *freedom and civil liberties*. At the same time, vaccination uptake increased.

While these are correlations and not to be interpreted as causal relationships, these findings may indicate that these policies might have been an essential factor in attenuating vaccination hesitancy—due to either the imposed restrictions for the unvaccinated or the decreased attention to medical concerns.

However, while vaccination hesitancy decreased, the discourse, which was connected to more negative than positive sentiments from the start, did not become more positive but, in fact, became more emotional.

These tendencies give reason to investigate the potential side effects of policy actions in more detail. In particular, it is essential to closely monitor whether policies, while increasing vaccination uptake, may simultaneously deepen the rifts between proponents and opponents of vaccination. Monitoring such data in real time can help identify rising tensions early and enable timely communicative responses such as tailored, audience-specific messaging that addresses specific concerns without legitimizing misinformation or undermining public health goals. Moreover, tracking the evolution of public concerns can help policymakers identify which fears require immediate attention, strategically time interventions, fine-tune policy designs to minimize unnecessary backlash, and detect emerging misinformation trends early. In addition, we found that the situation in other countries received considerable attention during all phases of the pandemic (RQ 3; Table 4). This suggests that policies and debates in other countries may strongly influence citizens’ opinions and behaviors. Citizens seek orientation beyond the borders of their country. This finding indicates the need for international solutions and cooperation.

For other vaccines, Zürcher et al [50] found German-speaking regions in Switzerland to be more vaccine hesitant than other Swiss regions. Among the possible factors contributing to this effect, they listed social influences, confidence in vaccination, or logistical barriers. In the case of the relatively high COVID-19 vaccine hesitancy among the German-speaking population compared to that in other European countries, logistical barriers logistical barriers seem to be a less likely explanation than social influences or/and a general lacking confidence in vaccination seem to be a less likely explanation than social influences as the low COVID-19 vaccination rates cannot be explained by a lack of vaccines alone and Germany was found to have had the most well-organized and sustained protest movement against COVID-19 measures in Europe (*Querdenker*), whereas Austria also faced protests [51]. Switzerland generally has a strong focus on individual liberties. The influence of German-speaking media across borders may have amplified skeptical narratives compared to French- or Italian-speaking regions.

A key challenge for policymakers is balancing public health measures with respect for individual freedoms. While our study did not directly address this trade-off, monitoring public discourse can help policymakers recognize concerns and adjust their responses accordingly. Our findings show that vaccination debates became increasingly emotional, particularly regarding tensions between public health and civil liberties. This underscores the need for communication strategies that go beyond factual information to address public anxieties through transparency, empathy, and engagement. In addition, understanding public concerns enables more targeted outreach, allowing for tailored messaging—such as evidence-based discussions with those with health concerns and community-focused incentives for free riders.

### Comparison With Prior Work

Several studies have investigated public opinion on COVID-19 vaccines through social media data, revealing both parallels to and differences from our findings. Hussain et al [52] reported mostly positive sentiments toward vaccines in the United Kingdom and United States, linked to development progress and availability. Similarly, Lyu et al [53] observed a dominance of trust and increasingly positive sentiments. In contrast, our dataset from German-speaking users exhibited more negative and emotional sentiments, especially over time. This divergence may partly stem from the different time frames studied; while the aforementioned studies’ analyses focused on earlier phases, ours extended into later, more polarized stages. Despite these differences, the identification by Lyu et al [53] of themes such as vaccine hesitancy and conspiracy theories closely aligns with the topics we found prevalent. Kwok et al [54] also identified attitudes toward vaccination, infection control, and misinformation as major topics in Australia, mirroring key concerns in our German-speaking sample, such as vaccine efficacy and civil liberties. Building on this, other studies have explored the role of external events in shaping public opinion. Hu et al [55] and Fazel et al [56] highlighted the influence of public announcements and social events on sentiment dynamics. We observed a similar pattern—in our data, shifts in sentiment aligned more strongly with policy changes than with pandemic infection waves. However, while Hu et al [55] documented a rise in positive sentiment following key events, we found that sentiments became increasingly negative and emotional—likely reflecting regional differences and the intensifying nature of policy restrictions in the DACH region. Focusing specifically on vaccine hesitancy, Nyawa et al [57] observed widespread distrust toward governments and concerns about vaccine efficacy and safety. Although our analysis included both hesitant and nonhesitant voices, these themes of efficacy and safety were similarly central to discussions in the German-speaking Twitter community. Closely related to our work, Bonnevie et al [25] and Herrera-Peco et al [27] identified health-related risks, vaccine safety, and conspiracy theories as dominant antivaccine narratives. These issues were equally salient in our dataset, with particular emphasis on concerns over vaccine side effects. Finally, Doogan et al [58] demonstrated that support for nonpharmaceutical interventions varied across countries and was not consistently tied to infection rates. This closely parallels our own finding—public discourse shifts correlated more with policy measures than with the actual pandemic progression. Their deeper analyses showed that less restrictive interventions tended to receive broader support, underlining the importance of designing policies that are sensitive to citizens’ concerns and perceptions.

To the best of our knowledge, our study is the first to analyze the evolution of vaccination discourse specifically in the German-speaking Twitter community and its relationship to policy phases, offering new insights into public concerns in later, more contentious stages of the pandemic.

### Limitations and Future Work

Our analysis has several limitations. First, Twitter users are not representative of the entire population. Therefore, analyzing tweets can serve to analyze fluctuations and tendencies but should not be interpreted as a representation of general public opinion. In addition, the precise method used by Twitter to sample 1% of their tweets randomly for API access is unknown. Therefore, the randomness of the sample cannot be guaranteed [59]. We relied on the language tag provided by Twitter to filter tweets in the German language. However, in principle, members of the German-speaking population may also tweet in languages other than German. Similarly, users tweeting in German do not necessarily have to be located in German-speaking countries. Still, we assume language to be the best proxy and assume at least a connection to German-speaking regions for users who tweet in the German language. In a similar vein, focusing on the German language excluded citizens in areas of the DACH countries that do not speak German (ie, certain regions in Switzerland). However, as previous research [50] has shown that vaccine uptake in Switzerland varies along linguistic lines, we argue that German serves as the best connecting factor among the 3 countries in our study. Moreover, the population in the 3 DACH countries is not distributed evenly. Assuming similar proportions of Twitter users in the 3 countries, most analyzed tweets would reflect German discourse as opposed to Swiss or Austrian. However, as the DACH countries faced comparable pandemic and policy phases, this did not affect the findings and conclusions of this study.

Second, while we tried to imbue as little previous knowledge into our analyses as possible, opting for a primarily data-driven approach, our analysis was influenced by the choice of policy events and the segmentation into pandemic and policy phases. We did not investigate other events beyond infection rates and policies that may influence or relate to the discourse, such as news or social media discussions.

Third, the assignment of themes was based on automatically generated topics but was still subjective. Different abstraction levels would have also been valid. The same applies to the generation of topics as such. The generated topics were not entirely selective (eg, topics in the *specific vaccines* cluster, such as the AstraZeneca topic, contained tweets that also discussed side effects, and vice versa). The same is true for the topic clusters discussing the effectiveness of vaccinations and the Omicron variant. To not produce too much noise, we decided to assign each tweet to the most probable cluster and not assign any cluster for low-confidence tweets. For future work, we will investigate the effects of assigning tweets to multiple clusters controlling for the noise generated by different thresholds and parameters and assessing topic cluster stability in different settings.

Finally, correlations regarding policy events and public attention give hints on possible connections, but no causal relations can be inferred in either direction. Our generated data can be analyzed further to draw more detailed insights on additional topics related to the formation and change in public opinion related to COVID-19 vaccinations. For example, while the attitudes and behaviors of influential individuals appeared to play an essential role in the public discourse on Twitter, it would be interesting to differentiate between different types of individuals, such as politicians or celebrities, advocates and opponents of vaccinations, and genuine versus false information in their statements to gain more insights on the role of issues of trust and misinformation. In addition, investigating cross-cultural differences in a comparative study across different regions or languages could help identify which discourse features correlate with actual vaccination rates in specific contexts, offering insights into how public sentiment and framing relate to vaccine uptake. Such analyses could also reveal regional differences in dominant concerns, sentiment trends, and the effectiveness of public health messaging.

### Conclusions

We proposed a hybrid pipeline for semiautomatic analysis of web discourse data for monitoring public debates. This includes a weakly supervised approach for seed list generation that allows for topical relevance filtering inserting as little previous knowledge as possible. For the analysis of themes, we used state-of-the-art topic modeling techniques for structuring the large datasets and enabling more in-depth manual analyses.

We gained insights into the attention to the topic of vaccinations among the German-speaking population on Twitter, the salience and evolution of subtopics and themes over time, and the associated sentiments.

By investigating policy actions and organizing them into phases, we revealed possible relationships and interactions between the salience of specific themes in the public web discourse and policies. Our findings suggest that analyzing web discourse data can yield valuable insights for policymakers regarding topics of interest and attention to public concerns in highly dynamic contexts such as the COVID-19 pandemic. Web discourse can be a fruitful data source in addition to traditional survey data.

## Appendix

Multimedia Appendix 1 Detailed information on automatically generated seed terms, groupings of topics to themes, and extracted policy events for Germany, Austria and Switzerland.

## Abbreviations

API: application programming interface
DACH: Deutschland (Germany), Austria, and CH (Confoederatio Helvetica, Latin for Switzerland)
RKI: Robert Koch Institute
RQ: research question

## Data Availability

The datasets generated or analyzed during this study are not publicly available due to Twitter’s restrictions regarding content distribution as stipulated in the company’s terms of service but are available from the corresponding author on reasonable request.
