# Supplementary material for: Themes, Policies, and Attention Shifts Regarding COVID-19 Vaccinations in German-Speaking Regions: Infoveillance Study Using Tweets
Source: J Med Internet Res. 2025 Oct 31;27:e63909. doi: 10.2196/63909 (PMC12619015; doi:10.2196/63909)
Supplement: Multimedia Appendix 1 [file jmir_v27i1e63909_app1.docx]

## Appendix 1

Detailed information on automatically generated seed terms, groupings of topics to themes, and extracted policy events for Germany, Austria and Switzerland.

*Table S1. Similarity- and co-occurrence-based generated seed list for the seed term “Impfung” (vaccination). Similarities were computed using Fasttext.cc embeddings trained on Wikipedia and Common Crawl. All terms with a cosine similarity higher or equal to 0.6 were ranked by their number of co-occurrences with the seed. The top 30 constitute the final seed list to filter vaccination-related tweets. English translations are added for comprehensibility.*

| Seed Term | Translation |
| --- | --- |
|  |  |
| impfung | vaccination |
| impfen | to vaccinate |
| impfstoff | vaccine |
| geimpfte | (the) vaccinated |
| impfungen | vaccinations |
| infektion | infection |
| impfpflicht | compulsory vaccination |
| geimpft | vaccinated |
| impfschutz | immunization protection |
| impftermin | vaccination appointment |
| impfschäden | vaccination damages |
| impfschaden | vaccination damage |
| immunisierung | immunization |
| impfkampagne | vaccination campaign |
| masern | measles |
| impfnebenwirkungen | vaccination side-effects |
| impfling | freshly vaccinated person / seed chrystal |
| erstimpfung | primary vaccination |
| grippeimpfung | influenza vaccination |
| impftermine | vaccination appointments |
| impfreaktionen | reactogenicities |
| impfreaktion | reactogenicity |
| impf | vaccination- |
| auffrischungsimpfung | booster injection |
| zwangsimpfung | compulsory vaccination |
| impfaktion | vaccination event |
| schweinegrippe | swine flu |
| impfstoffs | vaccine (direct object) |
| grundimmunisierung | fundamental immunization |
| impfbereitschaft | vaccination willingness |

*Table S2. Mappings of topics to manually derived themes with associated tweet numbers and averaged relative sentiments. Topics were generated by applying BERTopic on a 1% sample of tweets filtered for timestamps ranging from 01.01.2020 - 31.01.2022, German language tags and the tweet texts containing any vaccination-related terms. Themes were derived by manual inspection of the topics.* Sentiments were extracted with the SentiStrength tool, summed and normalized by their number. *# Number of tweets; <3 average relative sentiment; Included topics: ranks of the topics regarding their frequencies in all tweets and their manually assigned labels.*

| Theme | # | <3 | Included topics |
| --- | --- | --- | --- |
| Freedom and civil liberties | 9975 | -0.06 | 8 ”Regulations”, 11 ”Lockdowns”, 13 ”Basic rights”, 17 ”Compulsory vaccination”, 19 ”Masks and mask mandate”, 23 ”Demonstrations and protests”, 21 ”Compulsory vaccinations at work”, 34 ”Compulsory vaccination II”, 46 ”Political parties on compulsory vaccination”, 50 ”Vaccination as solidarity”, 60 ”Vaccination as a personal free choice”, 79 ”Police and police officers”, 82 ”Travel”, 84 ”Nazis”, 86 ”Fascism, vaccination as suppression”, 94 ”Privileges for the vaccinated and enforcement of vaccinations”, 107 ”Compulsory vaccination compared to road safety”, 112 ”Concerts and musicians”, 117 ”Compulsory vaccination debate”, 118 ”Spahn’s compulsory vaccination statement”, 119 ”Restaurant visits”, 121 ”Footballers and professional athletes”, 125 ”Discrimination of unvaccinated persons”, 129 ”Corona policies”, 137 ”Democracy, dictatorship, society”, 144 ”Compulsory vaccinations for all” |
| Safety and side effects | 5437 | -0.17 | 16 ”Vaccinations for elderly people”, 25 ”Vaccinations for children: medical views”, 35 ”Deaths due to or with Corona vaccination”, 36 ”Risks for pregnant women and infertility”, 40 ”Immediate vaccination side-effects”, 48 ”EMA”, 49 ”Side effects and risks with and without vaccination”, 63 ”Deaths after vaccination”, 65 ”Myocarditis risk after vaccination”, 69  ”AstraZeneca for specific age groups”, 76 ”Vaccination side effects”, 83 ”AstraZeneca vaccination stop”, 91 ”Safety of vaccinations”, 104 ”Effects of vaccinations on the menstrual cycle”, 109 ”Myocarditis risks”, 110 ”Pregnancy and risks”, 127 ”Side effects of vaccinations II”, 134 ”EMA  recommendations and authorizations”, 142 ”Development of vaccines and their tests”, 147 ”Allergies and allergic reactions” |
| Effectiveness | 5040 | -0.09 | 10 ”Duration of vaccine protection”, 12 ”Mutations due to vaccinations, spread of the virus when vaccinated”, 20 ”Vaccine efficacy for Omicron”,  27 ”Immune system”, 32 ”vaccination protection”, 98 ”Vaccination protection and efficacy”, 115 ”Number of vaccinated people in hospitals”, 116  ”Infections after being vaccinated”, 136 ”Anecdotes of vaccinations and infections”, 140 ”Virus variants and mutations” |
| Mobilization | 4982 | 0.12 | 5 ””Do (not) get vaccinated””, 29 ”#AllesInDenArm”, 41 ”Appeals to get vaccinated”, 47 ””I am vaccinated””, 54 ””I will not be vaccinated””, 68 ”Opinion about own vaccination”, 70 ”Congratulations to being vaccinated”, 71 ”Communication of free vaccination appointments”, 80 ””Got the second vaccination””, 85 ”Booking of vaccination appointments”, 89 ”Disputations between vaccinated and unvaccinated persons”, 97 ”Vaccination appointments for children”, 108 ”Vaccination status updates”, 124 ”Personal reasons for or against getting vaccinated”, 131 ”Disputes”, 141 ”Booster shots”, 143 ”Calls to sign petitions” |
| Details of the vaccination campaign | 2730 | 0.20 | 14 ”Practical implementation”, 33 ”Vaccine purchase EU”, 58 ”Costs, monetary incentives and penalties”, 59 ”Priority groups”, 64 ”Bratwurst  incentives”, 66 ”Who pays”, 90 ”Apps and digital vaccination certificates”, 120 ”Booked and free vaccination appointments” |
| Conspiracy theories | 1601 | -0.18 | 15 ”Propaganda and fake news”, 37 ”Bill Gates and vaccinations”, 74 ”Chips and implants” |
| Country comparisons | 6229 | -0.13 | 4 ”Situation in Germany (also in comparison with other countries)”, 7 ”Corona in Israel”, 30 ”Austria”, 43 ”Russia”, 61 ”Africa”, 62 ”Italy”, 67  ”France”, 73 ”Portugal”, 75 ”Vaccination of children in specific regions”, 77 ”China”, 88 ”Patent clearance”, 92 ”Great Britain”, 105 ”Israel”, 106 ”Global distribution of vaccines”, 126 ”Gibraltar”, 138 ”Switzerland” |
| Influential individuals | 4471 | -0.08 | 3 ”(prominent) vaccinated and unvaccinated men”, 24 ”Merkel”, 31 ”Lauterbach”, 39 ”Kimmich”, 55 ”Trump and Biden”, 72 ”Soeder”, 96 ”Sucharit Bhakdi”, 111 ”Kubicki”, 128 ”Politicians”, 149 ”BioNTech’s founder” |
| Specific vaccines | 3276 | 0.01 | 9 ”AstraZeneca vaccine”, 26 ”mRNA / gene-based vaccines”, 42 ”Sputnik vaccine”, 56 ”BioNTech”, 81 ”Novavax vaccine”, 100 ”Johnson & Johnson vaccine”, 101 ”Moderna vaccine”, 130 ”AstraZeneca vaccine II”, 145 ”J&J vaccine” |
| Data about the pandemic | 754 | 0.08 | 44 ”Statistics and headlines”, 78 ”Statistics about vaccination statuses”, 113 ”Statistics about the number of vaccinations”, 133 ”Statistics about  vaccination rates in Germany” |

*Table S3. List of policy events related to vaccinations manually extracted from the listed source websites with mappings to pandemic phases as classified by the Robert Koch Institute and manually assigned policy phases: Germany.*

| Date | Event | Source | Pandemic phase | Policy Phase |
| --- | --- | --- | --- | --- |
| 09/11/2020 | Leopoldina publishes position paper on vaccine distribution | https://www.leopoldina.org | Wave 2 | I |
| 17/12/2020 | First publication of the vaccination  recommendation (STIKO) | https://edoc.rki.de/handle/176904/7579 | Wave 2 | II |
| 18/12/2020 | Vaccination sequence published in the Federal Gazette | https://www.bundesanzeiger.de | Wave 2 | II |
| 21/12/2020 | Authorization of BioNTech vaccine | https://impfdashboard.de | Wave 2 | II |
| 27/12/2020 | Vaccination start | https://impfdashboard.de | Wave 2 | II |
| 06/01/2021 | Authorization of Moderna vaccine | https://impfdashboard.de | Wave 2 | II |
| 29/01/2021 | Authorization of AstraZeneca vaccine | https://impfdashboard.de | Wave 2 | II |
| 11/03/2021 | Authorization of Johnson & Johnson vaccine | https://impfdashboard.de | Wave 3 | II |
| 15/03/2021 | Halt of AstraZeneca vaccinations due to safety concerns | https://www.bundesregierung.de | Wave 3 | II |
| 25/03/2021 | Resumption of AstraZeneca vaccinations | https://www.bundesregierung.de | Wave 3 | II |
| 07/04/2021 | Nationwide vaccination in doctors’ offices | https://impfdashboard.de | Wave 3 | II |
| 06/05/2021 | Nationwide suspension of priority groups for AstraZeneca | https://www.ndr.de | Wave 3 | III |
| 28/05/2021 | Authorization of BioNTech vaccine  for youth | https://investors.biontech.de | Wave 3 | IV |
| 07/06/2021 | Official end of vaccination priority  groups | https://www.tagesschau.de | Wave 3 | IV |
| 23/08/2021 | Implementation of 3G Rule restricting access to facilities for unvaccinated and untested individuals | https://www.bundesregierung.de | Wave 4 | IV |
| 05/11/2021 | Health ministry decides on offering  booster shots | https://www.bundesregierung.de | Wave 4 | V |
| 26/11/2021 | Authorization of BioNTech vaccine  for children | https://www.pei.de | Wave 4 | V |
| 01/12/2021 | Vaccination stop for AstraZeneca  vaccine | https://www.zusammengegencorona.de | Wave 4 | V |
| 10/12/2021 | Adoption of facility-based mandatory vaccination | https://www.bgbl.de | Wave 4 | V |
| 21/12/2021 | STIKO recommends shorter time span before booster shot | https://www.rki.de | Wave 4 | V |
| 21/12/2021 | EU Commission decides on limited  validity of vaccination certificates | http://data.europa.eu | Wave 4 | V |
| 22/02/2022 | Authorization of Novavax vaccine | https://www.pei.de | Wave 5 | V |

*Table S4. List of policy events related to vaccinations manually extracted from the listed source websites with mappings to pandemic phases as classified by the Robert Koch Institute and manually assigned policy phases: Austria.*

| Date | Event | Source | Pandemic phase | Policy Phase |
| --- | --- | --- | --- | --- |
| 25/11/2020 | Chancellor’s office statement on ethical issues of vaccination published | https://www.bundeskanzleramt.gv.at | Wave 2 | II |
| 21/12/2020 | Authorization of BioNTech vaccine | https://www.basg.gv.at | Wave 2 | II |
| 26/12/2020 | Publication of priority groups | https://web.archive.org | Wave 2 | II |
| 27/12/2020 | Start of vaccinations | https://www.wienerzeitung.at | Wave 2 | II |
| 06/01/2021 | Authorization of Moderna vaccine | https://www.basg.gv.at | Wave 2 | II |
| 28/01/2021 | Regulation on the procedure of  Corona vaccination enters into effect | https://www.ots.at | Wave 2 | II |
| 29/01/2021 | Authorization of AstraZeneca vaccine | https://www.basg.gv.at | Wave 2 | II |
| 01/02/2021 | Vaccination plan published | https://www.sozialministerium.at | Wave 2 | II |
| 11/03/2021 | Authorization of Johnson & Johnson vaccine | https://www.basg.gv.at | Wave 3 | II |
| 25/05/2021 | BioNTech recommended for persons  older than 11 | https://www.basg.gv.at | Wave 3 | IV |
| 23/07/2021 | Moderna recommended for persons  older than 11 | https://www.basg.gv.at | Summer plateau 2021 | IV |
| 08/10/2021 | BioNTech booster recommended for from 6 months after second jab | https://www.basg.gv.at | Wave 4 | IV |
| 23/10/2021 | Introduction of lockdown for the unvaccinated upon reaching 600 patients in intensive care | https://www.wienerzeitung.at | Wave 4 | IV |
| 29/10/2021 | Moderna booster recommended for from 6 months after second jab | https://www.basg.gv.at | Wave 4 | IV |
| 15/11/2021 | Nationwide lockdown for the unvaccinated | https://www.wienerzeitung.at | Wave 4 | V |
| 25/11/2021 | Authorization of BioNTech vaccine  for children aged 5 - 11 | https://www.basg.gv.at | Wave 4 | V |
| 17/12/2021 | Johnson & Johnson booster recommended for from 6 months after second jab | https://www.basg.gv.at | Wave 4 | V |
| 20/12/2021 | Authorization of Novavax vaccine | https://www.basg.gv.at | Wave 4 | V |
| 20/01/2022 | Introduction of compulsory vaccinations for all citizens older than 17 | https://www.wienerzeitung.at | Wave 5 | V |

*Table S5. List of policy events related to vaccinations manually extracted from the listed source websites with mappings to pandemic phases as classified by the Robert Koch Institute and manually assigned policy phases: Switzerland.*

| Date | Event | Source | Pandemic phase | Policy Phase |
| --- | --- | --- | --- | --- |
| 17/12/2020 | Vaccination sequence published by the BAG | https://www.bag.admin.ch | Wave 2 | II |
| 19/12/2020 | Authorization of BioNTech vaccine | https://www.admin.ch | Wave 2 | II |
| 23/12/2020 | Start of vaccinations | https://www.srf.ch | Wave 2 | II |
| 12/01/2021 | Authorization of Moderna vaccine | https://www.swissmedic.ch | Wave 2 | II |
| 13/01/2021 | Vaccination costs covered by statutory health insurance approved | https://www.bag.admin.ch | Wave 2 | II |
| 21/01/2021 | Zurich is one of the first regions to  start involving primary care doctors | https://telebasel.ch | Wave 2 | II |
| 03/02/2021 | Swissmedic requests further data for approval for AstraZeneca | https://www.swissmedic.ch | Wave 2 | II |
| 22/03/2021 | Authorization of Johnson & Johnson  vaccine | https://www.swissmedic.ch | Wave 3 | II |
| 22/04/2021 | Work on international vaccination  certificate begins | https://www.bag.admin.ch | Wave 3 | III |
| 01/06/2021 | Those who have recovered should  also be vaccinated | https://www.srf.ch | Wave 3 | IV |
| 04/06/2021 | Legal basis for issuance of vaccination certificates created | https://www.bag.admin.ch | Wave 3 | IV |
| 26/10/2021 | Booster vaccination recommended  for persons older than 65 | https://www.bag.admin.ch | Wave 4 | IV |
| 04/11/2021 | Authorization of AstraZeneca not  further pursued | https://www.swissmedic.ch | Wave 4 | V |
| 26/11/2021 | Booster vaccination recommended  for the general population | https://www.bag.admin.ch | Wave 4 | V |
| 10/12/2021 | Authorization of BioNTech vaccine for children | https://www.bag.admin.ch | Wave 4 | V |
| 21/12/2021 | Recommendation to shorten the time until the booster vaccination | https://www.bag.admin.ch | Wave 4 | V |
